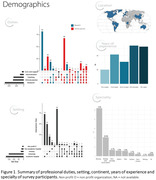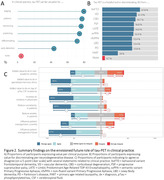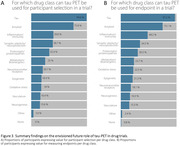# Experts envision a valuable role for tau‐PET in clinical practice and drug trials

**DOI:** 10.1002/alz.091257

**Published:** 2025-01-09

**Authors:** Marie R. Vermeiren, Ismael Luis Calandri, Wiesje M. van der Flier, Elsmarieke van de Giessen, Rik Ossenkoppele

**Affiliations:** ^1^ Department of Radiology & Nuclear Medicine, Amsterdam UMC, Amsterdam Netherlands; ^2^ Alzheimer Center Amsterdam, Amsterdam UMC, Amsterdam Netherlands; ^3^ Fleni, Buenos Aires, Buenos Aires Argentina; ^4^ Clinical Memory Research Unit, Lund University, Lund Sweden

## Abstract

**Background:**

Recent advancements in Alzheimer’s disease (AD) biomarker research and AD drug trials prompt reflection on the value and appropriate use of tau‐PET in future clinical practice and trials. We therefore conducted a survey among dementia and PET experts worldwide to investigate how they envision the future role of tau‐PET in clinical practice and trials.

**Method:**

An online survey was distributed to dementia clinicians and researchers who were invited to participate through personalized emails, social media channels and/or presentations at relevant conferences. With this approach we intended to recruit participants from different countries with diverse backgrounds and expertise. The survey questions explored experts’ opinions on the value of tau‐PET in clinical practice and in drug development and trials. We used a mix of multiple choice questions, statements with a 5‐point Likert scale (“strongly disagree” to “strongly agree”) and a few open questions.

**Result:**

In total 269 dementia experts, comprising 144 clinicians and 121 researchers, covering six continents completed the survey (Figure 1). The vast majority (90%) fosters a positive attitude on the added value of tau‐PET in clinical practice, particularly for staging, diagnosing, monitoring and prognostication in a cognitively impaired memory clinic population (Figure 2). When confronted with clinical case vignettes, our findings suggest that a tau‐PET scan is perceived particularly useful in patients with an atypical presentation (78%) or suspicion of mixed pathology (66%) and less useful in a typical AD case (25%). Experts are confident that a tau‐PET scan could influence patient management in current practice (median 4 “agree” [IQR 4‐5]) and this would increase when effective disease‐modifying treatments are available (median 4 “agree” [IQR 3‐4]) (Figure 2). Experts anticipate an important role for tau‐PET for participant selection (76‐100%) and measuring endpoints (75‐97%), in both anti‐amyloid and anti‐tau drug trials (Figure 3).

**Conclusion:**

Our global survey shows that dementia experts envision an important role for tau‐PET in the future, both in clinical practice and in drug trials.